# Sodium Intake and Osteoporosis Risk: Evidence From NHANES and Mendelian Randomization Analysis

**DOI:** 10.1002/fsn3.72147

**Published:** 2026-09-04

**Authors:** Yunxiao Ji, Zibo Fan, Rugang Zhao, Changsong Zhao, Qiang Zhang, Xinbao Wu

**Affiliations:** ^1^ Beijing Jishuitan Hospital Capital Medical University Beijing China; ^2^ Beijing Ditan Hospital Capital Medical University Beijing China; ^3^ Department of Orthopaedic Trauma Beijing Jishuitan Hospital, Capital Medical University Beijing China

**Keywords:** Mendelian randomization analysis, National Health and Nutrition Examination Survey, osteoporosis, sodium intake

## Abstract

Osteoporosis remains a critical public health challenge worldwide, particularly for older adults. Dietary sodium intake is considered a potentially modifiable factor related to bone mineral density (BMD) and osteoporosis risk, but existing findings remain inconsistent, and large‐scale evidence is limited. This study combines cross‐sectional analysis from the National Health and Nutrition Examination Survey (NHANES) with Mendelian randomization (MR) to investigate the association and potential causal relationship between sodium intake and osteoporosis. Drawing on NHANES data from the 2005–2010, 2013–2014, and 2017–2018 cycles (*n* = 3350), we applied weighted multivariable logistic regression to evaluate the association between sodium intake and osteoporosis risk. Leveraging sodium metabolism‐related single‐nucleotide polymorphisms (SNPs) as instrumental variables, we performed two‐sample Mendelian randomization (MR) analysis. MR estimates were derived using inverse‐variance weighting (IVW), MR‐Egger regression, and weighted median methods, with robustness assessed using pleiotropy tests and leave‐one‐out sensitivity analysis. Cross‐sectional analysis showed that each 1 mg/day increase in two‐day average dietary sodium intake was associated with approximately 0.011% lower odds of osteoporosis (OR = 0.99989, 95% CI: 0.99979–1.0000). Compared with the lowest quartile (Q1), participants in Q3 (OR = 0.48, 95% CI: 0.30–0.78, *p* = 0.004) and Q4 (OR = 0.51, 95% CI: 0.28–0.93, *p* = 0.028) had lower odds of osteoporosis (*p*‐trend < 0.05). MR analysis using the IVW method provided genetic evidence supporting a potential inverse association (OR = 0.51, 95% CI: 0.28–0.95), although the MR‐Egger estimate was nonsignificant and had a wide confidence interval (*p* = 0.312). These findings support a potential inverse association between sodium intake and osteoporosis risk in this study population. Further prospective and experimental studies are needed to validate these findings, clarify the underlying mechanisms, and determine whether they have any implications for dietary recommendations.

## Graphical abstract

This study combined cross‐sectional data from the National Health and Nutrition Examination Survey (NHANES) with Mendelian Randomization (MR) analysis to systematically investigate the relationship between sodium intake and osteoporosis. The results showed that within a specific intake range (> 6150 mg/day), sodium intake was significantly negatively associated with osteoporosis risk. Furthermore, MR analysis supported a potential causal protective effect. This research provides new scientific evidence for dietary prevention of osteoporosis.

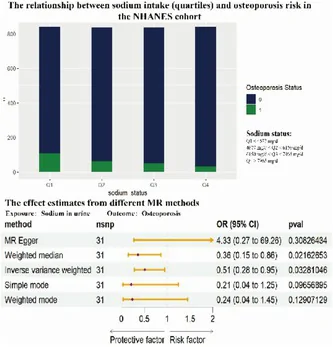

**Abbreviations**
95% CI: 95% confidence interval
BMI: body mass index
BPXD: diastolic blood pressure
BPXS: systolic blood pressure
CDC: the Centers for Disease Control and Prevention
ERB: research ethics review board
FNDDS: nutrient database for dietary studies
GWAS: genome‐wide association studies
HDL: high‐density lipoprotein
IV: instrumental variables
IVW: inverse‐variance weighted
LDL: low‐density lipoprotein
MR: the Mendelian randomization
NCHS: the National Center for Health Statistics
NHANES: The National Health and Nutrition Examination Survey
OR: odds ratio
SNPs: single nucleotide polymorphisms
Sod blood: serum sodium
TC: total cholesterol
TG: triglycerides

## 1 Introduction

Osteoporosis is characterized by decreased bone mineral density and destruction of bone microarchitecture (Nih Consensus Development Panel on Osteoporosis Prevention, Diagnosis, and Therapy 2001), resulting in a significant increase in fracture risk and representing an important public health problem worldwide. Epidemiological surveys indicate that osteoporosis affects millions of people globally, including an estimated 10 million individuals aged over 50 years in the United States (Clynes et al. 2020). Among the various factors related to osteoporosis, sodium intake has attracted growing interest as a potentially modifiable dietary factor, with evidence linking high sodium consumption to reduced bone mineral density (BMD). However, the relationship between sodium intake and BMD remains controversial, and further research is required to clarify its direction, mechanisms, and public health relevance.

Pioneering animal studies by Goulding et al. in the 1980s first demonstrated that excessive sodium intake accelerates urinary calcium excretion, thereby elevating osteoporosis risk. Building on this, a clinical cohort study of postmenopausal Korean women by Park et al. (2015) reported a positive association between high sodium consumption and elevated bone turnover markers. Conversely, other studies have failed to establish a clear link between sodium intake and bone health outcomes. For instance, Natri et al. (2005) conducted a trial in young healthy participants and found that a 7‐week sodium intake reduction showed no significant impact on bone metabolism markers. Other studies have suggested that a low‐salt diet can lead to a negative balance of trace elements such as calcium and magnesium, leading to an increased risk of osteoporosis (DiNicolantonio et al. 2018).

The heterogeneity of these findings may stem from methodological limitations, insufficient sample sizes, short follow‐up periods, and population heterogeneity. To address these research gaps, this study employed a multi‐faceted research design: first, large‐scale cross‐sectional data from the nationally representative NHANES were used to examine the association between sodium intake and osteoporosis; subsequently, MR was applied to explore the potential causal relationship using genetic variants as instrumental variables.

The NHANES is a cross‐sectional study conducted by the National Center for Health Statistics (NCHS), using a multi‐stage, stratified sampling design to assess the nutritional and health status of the U.S. population (Yuan et al. 2024). The dataset provides high‐quality, large‐sample, nationally representative data. MR uses genetic variants as instrumental variables. Because genetic variants are randomly assigned during meiosis and are generally less affected by environmental factors, MR can help reduce confounding and reverse causation in observational research (Verduijn et al. 2010; Lawlor et al. 2007).

This study has theoretical and public health relevance. It explores the association between sodium intake and osteoporosis risk and may help generate hypotheses for future osteoporosis prevention research. However, given the observational nature of the NHANES analysis and the proxy exposure used in MR, the findings should not be interpreted as direct dietary recommendations.

## 2 Methods

### 2.1 General Study Design

The study combines NHANES observational analysis with MR to assess the association and potential causal relationship between sodium intake and osteoporosis risk. The first component leveraged the NHANES database and used logistic regression with confounder adjustment to analyze the association between sodium intake and osteoporosis risk. The second component used two‐sample MR analysis based on pooled data from genome‐wide association studies (GWAS). Single Nucleotide Polymorphisms (SNPs) significantly associated with urinary sodium were selected as instrumental variables. The primary analysis was performed using the Inverse‐Variance Weighted (IVW) method and was supported by sensitivity analysis using the Weighted Median and MR‐Egger regression methods.

### 2.2 Observational Study Sample Population in NHANES

#### 2.2.1 Data Source and Cohort Profile

**FIGURE 1:**
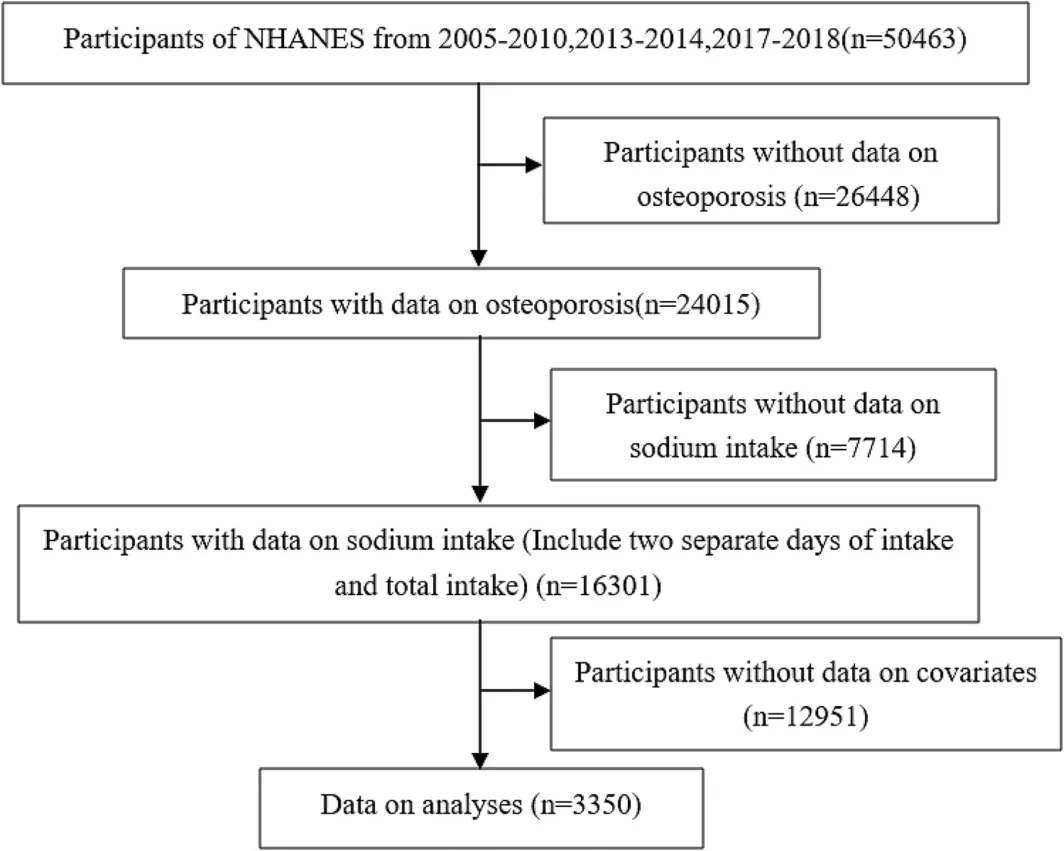
Flowchart of eligible patient screening.

The dataset employed in this research was obtained from publicly available data from the 2005–2010, 2013–2014, and 2017–2018 cycles of NHANES. NHANES, overseen by the Centers for Disease Control and Prevention (CDC) and the NCHS, provides nationally representative data on U.S. population health and nutrition. It collects information through standardized interviews, physical examinations, and laboratory tests (Paulose‐Ram et al. 2021). Eligibility criteria were as follows: (1) age ≥ 20 years; (2) Completion of two consecutive 24‐h dietary recall survey; (3) Participants who participated in the Osteoporosis Questionnaire; (4) No history of skeletal deformities or metabolic bone disorders (e.g., Paget's disease, osteogenesis imperfecta). Exclusion criteria: (1) ≥ 5% missing clinical data (e.g., incomplete dietary records, missing covariates); (2) Relevant confounders such as diseases that may lead to femoral head necrosis; (3) Conditions influencing bone metabolism or leading to secondary osteoporosis including chronic kidney disease (CKD stage 3 or above), hyperparathyroidism or hypoparathyroidism, Cushing's syndrome, prolonged glucocorticoid use (> 3 months), rheumatoid arthritis, or other autoimmune disease requiring immunosuppression; (4) Long‐term use of medications affecting bone health, including: immunosuppressants (e.g., cyclosporine, methotrexate), anticonvulsants (e.g., phenytoin, carbamazepine), and proton‐pump inhibitors (PPIs) for more than 1 year; (5) Significant trauma or fracture within the last 1 year (to avoid acute bone loss confounding factors); (6) Non‐ambulatory status or severe mobility limitations (e.g., wheelchair dependence); (7) extreme dietary patterns (e.g., unsupplemented vegetarianism, severe malnutrition). A total of 50,463 participants were initially identified, and 3350 subjects were ultimately inclued in this study (the specific exclusion process is shown in Figure 1). The NCHS Research Ethics Review Board (ERB) approved the data collection procedures (Protocol #2005‐06, Protocol #2011‐17, and Protocol #2018‐01), and all participants signed an informed consent form.

#### 2.2.2 Variable Definition and Statistical Analysis Methods

Sodium intake was quantified using 2‐day 24‐h dietary recall data from NHANES, with calculations performed via the USDA Food and Nutrient Database for Dietary Studies (FNDDS). The FNDDS improves data accuracy through chemical analyses and standardized recipe coding, and daily sodium intake was quantified in mg/day. To analyze the dose‐response relationship between sodium intake and osteoporosis risk, total sodium intake was divided into four groups (Q1‐Q4) by quartiles, and the 2‐day average sodium intake was also analyzed as a continuous variable. In the NHANES cycles used in this study, BMD measurements were not available for all participants. To maximize sample size and maintain population representativeness, osteoporosis status was defined using OSQ060, which records self‐reported physician‐diagnosed osteoporosis (“Have you ever been told you have osteoporosis?”). This variable is collected using a standardized method documented in the NHANES protocol and has been used in recent NHANES‐based osteoporosis studies (Fu and Wang 2025). Nevertheless, this definition cannot replace BMD‐based diagnosis and may lead to misclassification or recall bias. This limitation is acknowledged in the Section 4.

During statistical analysis, the study fully considered the complex sampling design of NHANES, applying official sample weights, stratified variables, etc., to ensure the results were nationally representative of the U.S. population. The analysis employed a weighted logistic regression model adjusted for multiple variables. Furthermore, three levels of regression models were established: model 1 was uncorrected for any covariates and was used to calculate the crude odds ratio (OR) of sodium intake to osteoporosis; model 2 was corrected for the basic demographic variables including age and sex; model 3 incorporated additional adjustments for multiple variables, encompassing demographic characteristics (age, sex, marital status, education), lifestyle factors (smoking, sex, alcohol consumption), metabolic indicators (blood pressure, diabetes status, body mass index (BMI)), and all potential confounders comprising lipid profile (low‐density lipoprotein (LDL), triglyceride (TG), High‐density lipoprotein (HDL)) and serum uric acid levels. Continuous variables were expressed as odds ratios (ORs) and 95% confidence intervals (95% CIs), and the percentage change in risk corresponding to each 1 mg/day increase in sodium intake was calculated; categorical variable analyses were conducted using the lowest quartile group (Q1: sodium intake ≤ 4677 mg/day) as a reference, and OR and 95% CI were reported for the other groups. All statistical analyses were completed using the survey package in R 4.1.0 software, and the significance level was set at a two‐sided *p* < 0.05.

To improve methodological rigor, we used standardized methods for variable definition, data collection, and statistical analysis. Sodium intake was assessed based on the validated FNDDS database, and osteoporosis status was defined using a standardized NHANES questionnaire item for self‐reported physician‐diagnosed osteoporosis. Statistical analyses followed NHANES guidance by applying complex sampling weights and multivariable adjustment. This design supports population‐level inference, while the limitations of questionnaire‐based osteoporosis ascertainment and residual confounding are acknowledged.

### 2.3 Mendelian Randomization

#### 2.3.1 Study Design and Data Sources

Study design: A two‐sample MR analysis was performed to explore the potential causal relationship between sodium‐related traits and osteoporosis occurrence. Because GWAS data for dietary sodium intake were not available, urinary sodium was used as a surrogate exposure. Twenty‐four‐hours urinary sodium excretion is widely regarded as a reference method for assessing sodium intake at the population level (Wen et al. 2019; World Health Organization, n.d.). The International Consortium for Quality Research on Dietary Sodium/Salt (TRUE) has emphasized that 24‐h urine collection is the most reliable method for estimating daily sodium intake (Campbell et al. 2019), and previous validation studies support urinary sodium as a proxy for dietary sodium intake (Gong et al. 2022; Wielgosz et al. 2015). However, because individual‐level dietary sodium data were not available in the GWAS dataset, we could not directly calculate a Pearson correlation between dietary sodium intake and urinary sodium in the MR population. Therefore, urinary sodium was used as a biologically plausible proxy supported by previous validation studies, while this proxy assumption was acknowledged as a limitation. To investigate the effect of exposure on outcomes, MR analysis must satisfy three key assumptions: (1) genetic variants are associated with urinary sodium; (2) genetic variants are not associated with confounders; and (3) genetic variants influence osteoporosis only through urinary sodium (Larsson et al. 2023).

We obtained urinary sodium‐related genetic instrument from the Integrative Epidemiology Unit (IEU) in GWAS, which included 326,831 European individuals. SNPs suitable as instrumental variables (IV) were selected as follows: (1) Extract SNPs associated with urinary sodium as IV at the genome‐wide significance threshold (*p* < 5e^−8^). (2) Remove SNPs exhibiting linkage disequilibrium (defined as *r* ^2^ < 0.001 within a 10,000 kb window), and extract the remaining SNPs from the result dataset. (3) We calculated the *F*‐statistic for each SNP to quantify the strength of the exposure and exclude weak instruments (*F*‐statistic < 10). The *F*‐statistic for each SNP was derived as (β/SE)^2^. To assess the overall instrument strength, we computed the mean *F*‐statistic across all selected SNPs. The overall *F*‐statistic was 41.26 (all individual SNP *F*‐statistics > 10), indicating that the instrumental variables are sufficiently strong and not prone to weak instrument bias. Detailed *F*‐statistics for each of the 31 SNPs used as instrumental variables are provided in Table S1. Variables associated with osteoporosis also came from IEU, which included 212,778 European participants (3203 patients and 209,575 control participants) (Davies et al. 2018). GWAS data for both exposure and outcome traits in this study were sourced from the IEU OpenGWAS Platform. The raw data has been fully de‐identified and the platform has confirmed that the collection process complies with the ethical standards of the Declaration of Helsinki. In accordance with Article 89 of the European Union's General Data Protection Regulation (GDPR), secondary analysis of anonymized data does not require additional ethical approval.

#### 2.3.2 Statistical Analysis

Using the SNPs described above, we adopted the inverse variance‐weighted (IVW) method as the primary analytical approach, which offered the greatest statistical power while demonstrating insensitivity to horizontal pleiotropy. In order to further verify results robustness, the investigators added the following sensitivity analysis: (1) Pleiotropy evaluation: the potential horizontal pleiotropy was evaluated via the intercept term of MR‐Egger regression (Bowden et al. 2015); (2) Heterogeneity assessment: Cochran's Q test was employed to detect heterogeneity among instrumental variables (Burgess et al. 2013); (3) Validation of alternative methods, including weighted median estimator (WME), weighted mode, and simple mode, to reduce the risk of bias due to invalid instrumental variables or pleiotropies (Bowden et al. 2016); (4) Leave‐one‐out (LOO) analysis: By eliminating individual SNPs one by one and repeating the analysis, individual SNPs that may have an excessive impact on the results are identified (Burgess and Thompson 2017).

## 3 Results

### 3.1 General Characteristics of NHANES

**FIGURE 2:**
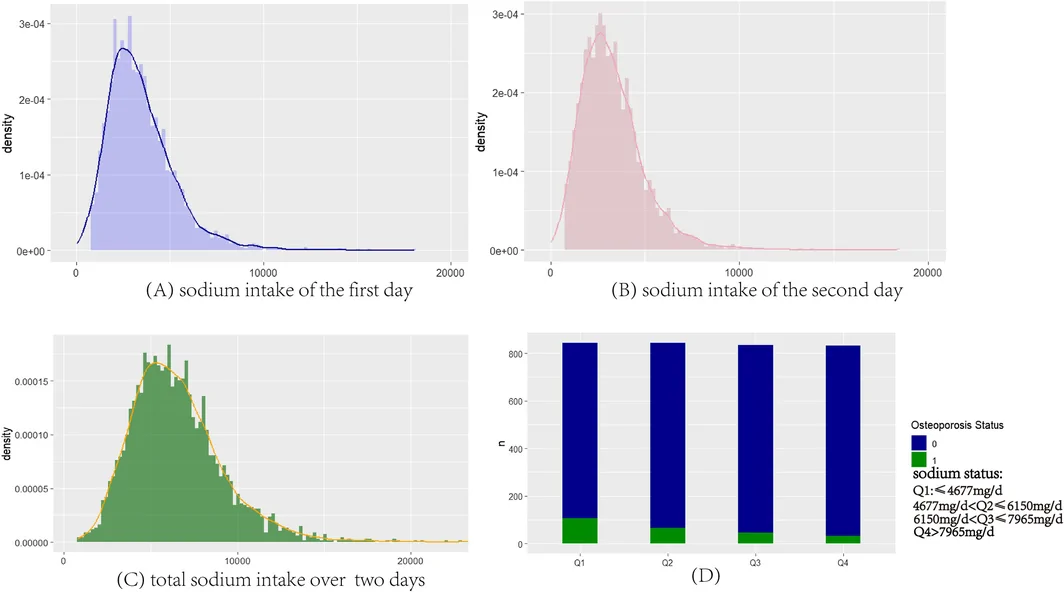
The distribution of sodium intake. The database surveyed sodium intake over 2 days. (A) Presents the intake distribution of the population on the first day; (B) Presents the intake distribution of the population on the first day; (C) Shows the distribution of total sodium intake over the 2 days; (D) Shows the relationship between different levels of sodium intake and osteoporosis. The sodium intake was divided into four levels (Q1, Q2, Q3, and Q4) based on quartiles. “0” represents no occurrence of osteoporosis, while “1” represents osteoporosis.

This study analyzed the baseline characteristics of 3350 participants based on NHANES data, divided into four groups Q1‐Q4 by sodium intake quartile (Q1: sodium intake ≤ 4677 mg/day; Q2: sodium intake: 4677 mg/day < Q2 ≤ 6150 mg/day; Q3: sodium intake: 6150 mg/day < Q3 ≤ 7965 mg/day; Q4: sodium intake: > 7965 mg/day). Overall, participants have a mean age of 55.17 (SD = 16.46) years, BMI of 29.02 (SD = 6.59), with 57.2% male and 42.8% female. Group differences were observed in ethnicity, education, and marital status. No significant intergroup difference was found in smoking history, whereas a significant difference existed in alcohol history (*p* < 0.001). Among the serological indicators, there was a difference in HDL between groups (*p* = 0.001), but no statistical difference was observed in TG and LDL levels (*p* > 0.05). For underlying diseases, the prevalence of hypertension and osteoporosis showed significant intergroup variation. See Table 1 for details. Figure 2 further illustrates the distribution of sodium intake, with the lowest sodium intake in the Q1 group and the highest in the Q4 group, indicating that sodium intake might have a critical role in bone health.

**TABLE 1:** Baseline characteristics of unweighted participants included in the analysis.

Variables	Overall (*n* = 3350)	Q1 (*n* = 843)	Q2 (*n* = 842)	Q3 (*n* = 834)	Q4 (*n* = 831)	*p*
Age (years)	55.17 ± 16.46	58.88 ± 15,48	55.90 ± 16.59	54.93 ± 16.53	50.91 ± 16.25	**< 0.001**
BMI (kg/m^2^)	29.02 ± 6.59	28.61 ± 6.33	29.20 ± 6.65	29.20 ± 6.65	29.08 ± 6.70	0.208
BPXS (mmHg)	125.06 ± 19.04	127.08 ± 20.39	125.06 ± 20.07	124.92 ± 18.77	123.16 ± 16.46	**< 0.001**
BPXD (mmHg)	68.5 7 ± 13.47	68.27 ± 13.55	67.59 ± 14.05	68.72 ± 12.85	69.72 ± 13.31	**0.011**
Serum uric acid (mg/dL)	335.30 ± 87.26	331.45 ± 86.60	334.93 ± 91.27	336.85 ± 87.43	338.04 ± 83.53	0.434
TG (mg/dL)	1.46 ± 0.77	1.49 ± 0.77	1.43 ± 0.73	1.50 ± 0.82	1.43 ± 0.76	0.103
LDL (mg/dL)	2.95 ± 0.97	2.98 ± 1.02	2.94 ± 0.96	2.95 ± 0.95	2.91 ± 0.94	0.528
HDL (mg/dL)	1.39 ± 0.43	1.41 ± 0.46	1.42 ± 0.44	1.37 ± 0.40	1.35 ± 0.40	**0.001**
TC (mg/dL)	5.00 ± 1.11	5.08 ± 1.17	5.02 ± 1.12	5.00 ± 1.09	4.91 ± 1.04	**0.023**
Sodium_intake_1st (mg/day)	3432.87 ± 1834.84	1922.80 ± 785.97	2833.89 ± 903.14	3666.84 ± 1114.12	5336.85 ± 2126.20	**< 0.001**
Sodium_intake_2nd (mg/day)	3264.97 ± 1711.68	2250.54 ± 1272.46	2971.95 ± 1305.66	3350.58 ± 1420.24	4505.01 ± 1936.06	**< 0.001**
Sodium total intake (mg/day)	6528.89 ± 2700.31	3605.39 ± 817.84	5419.98 ± 432.88	7013.56 ± 505.77	10131.78 ± 2266.21	**< 0.001**
Sod blood (mmol/L)	139.43 ± 2.49	139.56 ± 2.63	139.38 ± 2.41	139.42 ± 2.38	139.34 ± 2.55	0.312
Gender						
Male	1916 ± 57.2	364 ± 43.2	441 ± 52.4	505 ± 60.6	606 ± 72.9	**< 0.001**
Female	1434 ± 42.8	47 9 ± 56.8	401 ± 47.6	329 ± 39.4	225 ± 27.1	
Race (%)						
Mexican American	429 (12.8)	109 (12.9)	105 (12.5)	124 (14.9)	91 (11.0)	**0.028**
Other Hispanic	250 (7.5)	68 (8.1)	67 (8.0)	71 (8.5)	44 (5.3)	
Non‐Hispanic White	1867 (55.7)	441 (52.3)	470 (55.8)	462 (55.4)	494 (59.4)	
Non‐Hispanic Black	636 (19.0)	183 (21.7)	160 (19.0)	136 (16.3)	157 (18.9)	
Other race	168 (5.0)	42 (5.0)	40 (4.8)	41 (4.9)	45 (5.4)	
Education (%)						
Less than 9th grade	340 (10.1)	102 (12.1)	103 (12.2)	82 (9.8)	53 (6.4)	**0.001**
9‐11th grade (Includes 12th grade with no diploma)	617 (18.4)	175 (20.8)	156 (18.5)	141 (16.9)	145 (17.4)	
High school Graduate/GED or equivalent	870 (26.0)	217 (25.7)	228 (27.1)	200 (24.0)	225 (27.1)	
Some college or AA degree	965 (28.8)	227 (26.9)	223 (26.5)	260 (31.2)	255 (30.7)	
College graduate or above	557 (16.6)	121 (14.4)	132 (15.7)	151 (18.1)	153 (18.4)	
Don't know	1 (0.0)	1 (0.1)	0 (0.0)	0 (0.0)	0 (0.0)	
Marital (%)						
Married	1765 (52.7)	381 (45.2)	453 (53.8)	476 (57.1)	455 (54.8)	**< 0.001**
Widowed	303 (9.0)	128 (15.2)	73 (8.7)	63 (7.6)	39 (4.7)	
Divorced	474 (14.1)	141 (16.7)	118 (14.0)	97 (11.6)	118 (14.2)	
Separated	116 (3.5)	36 (4.3)	26 (3.1)	30 (3.6)	24 (2.9)	
Never married	397 (11.9)	96 (11.4)	96 (11.4)	91 (10.9)	114 (13.7)	
Living with partner	294 (8.8)	61 (7.2)	75 (8.9)	77 (9.2)	81 (9.7)	
Refused	1 (0.0)	0 (0.0)	1 (0.1)	0 (0.0)	0 (0.0)	
Cigarettes (%)						
Yes	1384 (41.3)	357 (42.3)	346 (41.1)	320 (38.4)	361 (43.4)	0.177
No	1966 (58.7)	486 (57.7)	496 (58.9)	514 (61.6)	470 (56.6)	
Alcohol (%)						
Yes	559 (16.7)	105 (12.5)	139 (16.5)	147 (17.6)	168 (20.2)	**< 0.001**
No	2791 (83.3)	738 (87.5)	703 (83.5)	687 (82.4)	663 (79.8)	
Hypertension (%)						
Yes	1461 (43.6)	420 (49.8)	362 (43.0)	364 (43.6)	315 (37.9)	**< 0.001**
No	1885 (56.3)	421 (49.9)	479 (56.9)	469 (56.2)	516 (62.1)	
Don't know	4 (0.1)	2 (0.2)	1 (0.1)	1 (0.1)	0 (0.0)	
Diabetes (%)						
Yes	502 (15.0)	147 (17.4)	111 (13.2)	134 (16.1)	110 (13.2)	0.086
No	2750 (82.1)	671 (79.6)	713 (84.7)	671 (80.5)	695 (83.6)	
Borderline	97 (2.9)	24 (2.8)	18 (2.1)	29 (3.5)	26 (3.1)	
Don't know	1 (0.0)	1 (0.1)	0 (0.0)	0 (0.0)	0 (0.0)	
Sodium status (%)						
Q1	843 (25.2)	843 (100.0)	0 (0.0)	0 (0.0)	0 (0.0)	**< 0.001**
Q2	842 (25.1)	0 (0.0)	842 (100.0)	0 (0.0)	0 (0.0)	
Q3	834 (24.9)	0 (0.0)	0 (0.0)	834 (100.0)	0 (0.0)	
Q4	831 (24.8)	0 (0.0)	0 (0.0)	0 (0.0)	831 (100.0)	
Osteoporosis status (%)						
Yes	248 (7.4)	106 (12.6)	65 (7.7)	45 (5.4)	32 (3.9)	**< 0.001**
No	3120 (92.6)	737 (87.4)	777 (92.3)	789 (94.6)	799 (96.1)	

*Note:* This table presents the baseline demographic, clinical, and lifestyle characteristics of participants stratified into four quartiles (Q1–Q4) based on sodium intake.

Abbreviations: BMI, body mass index; BPXD, diastolic blood pressure; BPXS, systolic blood pressure; HDL, high‐density lipoprotein; LDL, low‐density lipoprotein; Sod blood, serum sodium; TC, total cholesterol; TG, triglycerides. Bold values indicate statistically significant *p* values (*p* < 0.05).

### 3.2 Association Between Sodium Intake and Osteoporosis

Weighted multivariable logistic regression based on the complex NHANES sampling design showed an inverse association between sodium intake and osteoporosis risk in both continuous and categorical analyses (*p* < 0.05), although the effect size in the continuous‐variable analysis was small (Model1: β = −2.65 × 10^−4^; OR = 0.99974, 95% CI: 0.99965–0.9998; Model2: β = −1.17 × 10^−4^; OR = 0.99988, 95% CI:0.99979–1.0000; Model3: β = −1.14 × 10^−4^; OR = 0.99989, 95% CI:0.99979–1.0000). In the quartile‐based analysis, after adjustment for all covariates, higher sodium intake was associated with lower odds of osteoporosis. Compared with the lowest quartile group (Q1), the Q3 group (sodium intake: 6150 mg/day < Q3 ≤ 7965 mg/day) had a 52% reduction in osteoporosis (Model 3: OR = 0.48, 95% CI: 0.30–0.78, *p* = 0.004) and a 49% reduction in osteoporosis in Q4 (sodium intake: Q4: > 7965 mg/day) (OR = 0.51, 95% CI: 0.28–0.93, *p* = 0.028). See Table 2 for details.

**TABLE 2:** Association between weight‐adjusted sodium intake and osteoporosis.

Exposure	Model 1		Model 2		Model 3	
	OR (95% Cl)	*p*	OR (95% Cl)	*p*	OR (95% Cl)	*p*
Sodium intake (continues)	0.99974 (0.99965, 0.9998)	< 0.001	0.99988 (0.99979, 1.0000)	0.0171	0.99989 (0.99979, 1.0000)	0.0260
Sodium intake (quartile)						
Q1	Reference		Reference		Reference	
Q2	0.65 (0.43, 0.99)	0.04	0.86 (0.55, 1.34)	0.50	0.88 (0.57, 1.35)	0.55
Q3	0.28 (0.18, 0.45)	< 0.001	0.46 (0.29, 0.72)	0.001	0.48 (0.30, 0.78)	0.004
Q4	0.18 (0.11, 0.31)	< 0.001	0.51 (0.28, 0.93)	0.030	0.51 (0.28, 0.93)	0.028
Sodium Intake (Average of two days)	0.99944 (0.99929, 0.9996)	< 0.001	0.99979 (0.99960, 1.0000)	0.0303	0.99979 (0.99959, 1.0000)	0.0473

*Note:* Q1: sodium intake ≤ 4677 mg/day; Q2: sodium intake: 4677 mg/day < Q2 ≤ 6150 mg/day; Q3: sodium intake: 6150 mg/day < Q3 ≤ 7965 mg/day; Q4: sodium intake: > 7965 mg/day Model 1 adjusted for: none; Model 2 adjusted for: gender, age; Model 3 adjusted for: serum sodium, age, gender, alcohol, cigarettes, total cholesterol, diabetes, hypertension, systolic blood pressure, triglycerides, systolic blood pressure, diastolic blood pressure, low‐density lipoprotein cholesterol, high‐density lipoprotein cholesterol, serum uric acid and education, marital.

**FIGURE 3:**
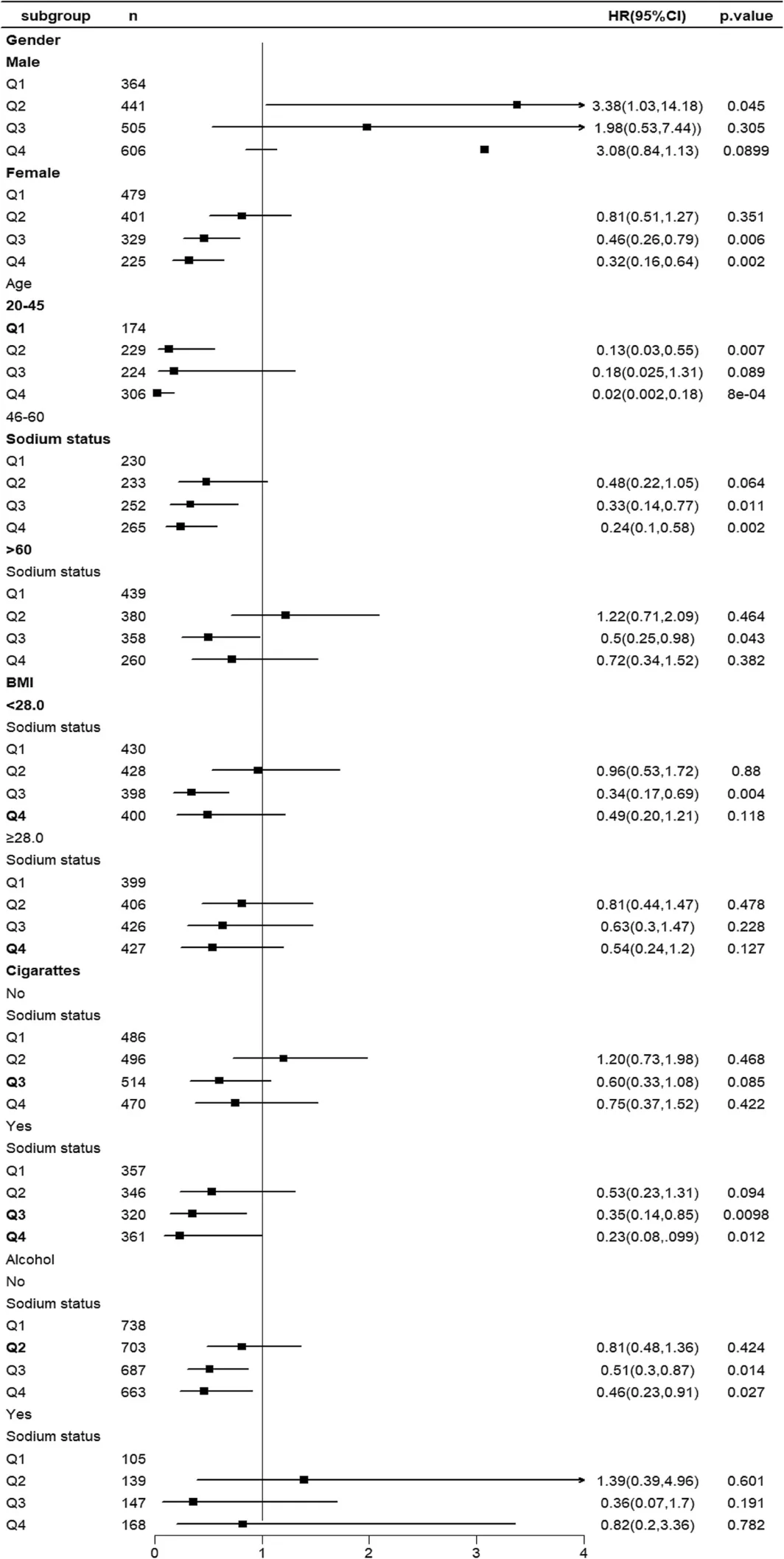
Subgroup analysis of sodium intake and health outcomes. Gender: The plot shows ORs for male and female participants across different sodium intake quartiles (Q1–Q4); Age: Participants are divided into two age groups: 20–45 and 46–60 years. Sodium status: Participants are categorized based on their sodium intake status (< 60, ≥ 60). BMI: Participants are grouped by BMI categories (< 28.0, ≥ 28.0); Cigarettes: Participants are divided into non‐smokers and smokers; Alcohol: Participants are categorized based on alcohol consumption (no, yes); Overall sodium intake: Participants are divided into quartiles based on their total sodium intake.

In this study, the association between sodium intake and osteoporosis risk was further evaluated by stratified analysis, and the results showed significant heterogeneity between different populations (Figure 3). In women, the risk of osteoporosis decreased significantly in a dose‐dependent manner with the increase of sodium intake, and the risk in the Q4 group (highest intake group) was significantly lower than that in the Q1 group (lowest intake group) by 68% (OR = 0.32, 95% CI: 0.16–0.64, *p* = 0.002). In males, this protective association lacked statistical significance (*p* > 0.05). Age‐stratified analysis showed that high sodium intake in young adults (20–45 years) in the Q3 group was significantly associated with a 98% reduced osteoporosis risk (OR = 0.02, 95% CI: 0.002–0.18, *p* = 8 × 10^−4^), but only moderate protective effects were observed in older adults over 60 years of age. Notably, moderate sodium intake (Q2 group) showed significant protective effect in the normal weight population with a BMI of < 28 (OR = 0.34, 95% CI: 0.17–0.69, *p* = 0.004). In addition, the risk was reduced by 65%–77% in the high‐sodium intake group in the smokers (*p* < 0.05), while drinking status did not significantly alter this association. These findings suggest that the protective effect of sodium intake on osteoporosis may be regulated by gender, age, and metabolic status, which provides an important basis for the development of individualized dietary prevention strategies for osteoporosis. Future studies need to further elucidate the molecular mechanism of high‐sodium diet on bone metabolism, especially the differential response mechanism in different populations.

### 3.3 Dose–Response Relationship Analysis

**FIGURE 4:**
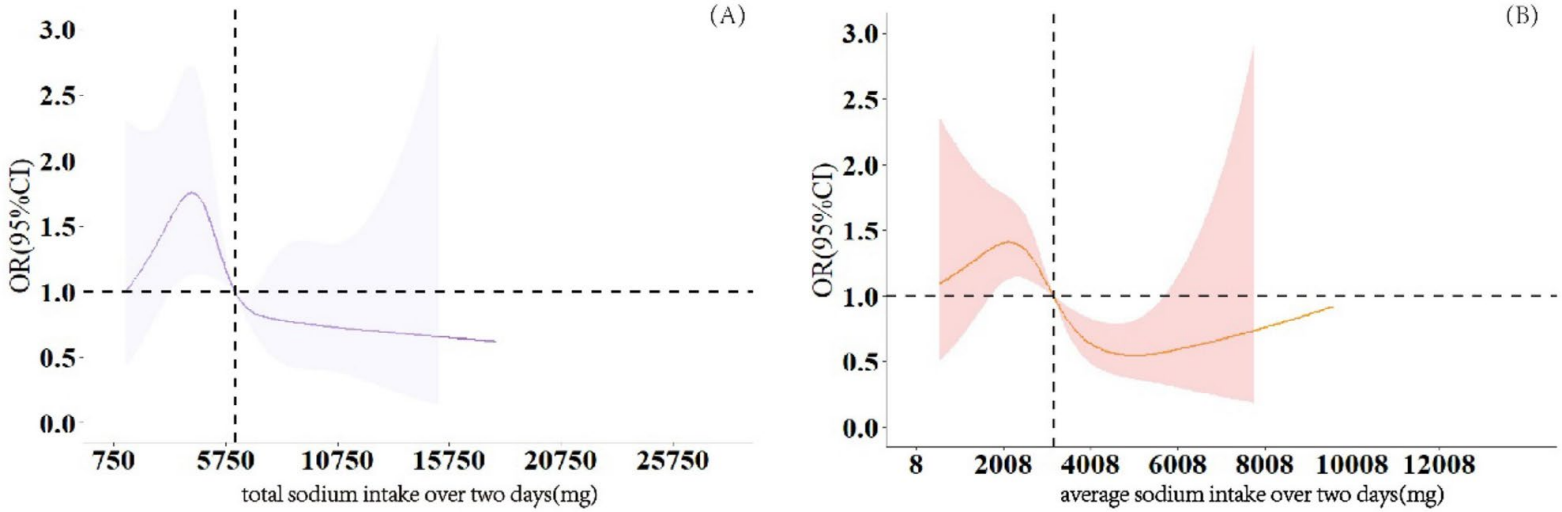
Dose–response relationship between sodium intake and the risk of osteoporosis. (A) Illustrates the relationship between total sodium intake and the risk of osteoporosis. The solid line in the graph represents the odds ratio (OR) with its 95% confidence interval (CI), and the shaded area indicates the range of the confidence interval. When total sodium intake exceeds 6156 mg, the OR falls below 1, suggesting that within this range, an increase in sodium intake may be associated with a reduced risk of osteoporosis.; (B) Depicts the relationship between the average sodium intake over 2 days and the risk of osteoporosis. Similarly, the solid line represents the OR with its 95% CI, and the shaded area shows the confidence interval. When the average sodium intake surpasses 3143.5 mg, the OR decreases again, indicating that within this range, an increase in sodium intake may be associated with a reduced risk of osteoporosis. *X*‐axis: Sodium intake (total sodium intake for Figure 4A, average sodium intake over 2 days for Figure 4B); *Y*‐axis: Odds ratio (OR) with 95% confidence interval (CI); Solid line: Estimated OR value; Shaded area: Range of the 95% CI; Dashed line: Reference line for OR = 1, indicating no significant association.

The dose–response relationship between total sodium intake, two‐day average sodium intake and bone mineral density was further analyzed by limiting cubic spline (RCS). For Figure 4, a significant overall association was observed between total sodium intake and osteoporosis risk (*p* = 0.0026), whereas the test for nonlinearity was nonsignificant (*p* = 0.128). At total sodium intake levels above approximately 6156 mg, the odds ratio decreased below 1, suggesting a lower estimated osteoporosis risk within this study population. Similarly, the 2‐day average sodium intake also showed a significant overall association (*p* = 0.0026), whereas the nonlinearity test was nonsignificant (*p* = 0.079). At average daily sodium intake levels above approximately 3143.5 mg/day, the odds ratio again decreased below 1. The apparent inflection points observed in the RCS analysis should be interpreted as study‐specific statistical features rather than clinically actionable intake targets.

### 3.4 MR of Sodium Intake and Osteoporosis

**FIGURE 5:**
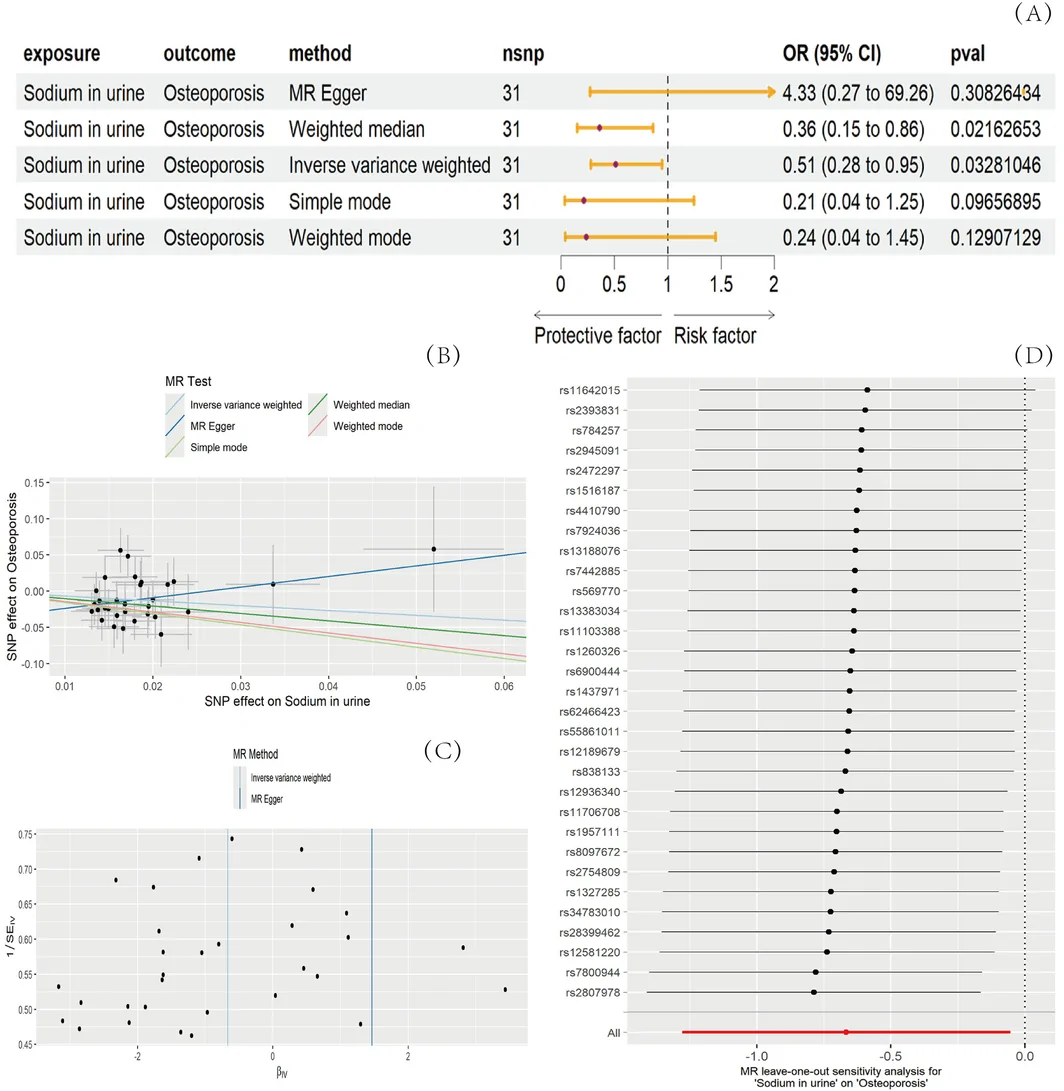
Mendelian randomization analysis of urinary sodium on osteoporosis risk: IVW and sensitivity methods. (A) Causal estimates of urinary sodium on osteoporosis using multiple Mendelian randomization (MR) methods. Key findings: (1) Inverse‐variance weighted (IVW) and weighted median methods showed a significant protective effect of higher urinary sodium levels against osteoporosis (IVW: OR = 0.51, 95% CI: 0.28–0.95, *p* = 0.033; weighted median: OR = 0.36, 95% CI: 0.15–0.86, *p* = 0.022). (2) MR‐Egger regression indicated a non‐significant positive association (OR = 4.33, *p* = 0.308), suggesting potential pleiotropy, though the intercept test was non‐significant (not shown). (B) MR sensitivity test. Scatter plot of SNP effects on urinary sodium (*x*‐axis) versus osteoporosis risk (*y*‐axis). Fitted lines represent MR methods: The negative slopes of IVW (blue) and weighted median (green) support a causal protective role of urinary sodium. (C) Funnel plot (symmetry test). Symmetrical distribution of SNP effects around the null line (*p* > 0.05) supports instrument validity. (D) Leave‐one‐out sensitivity analysis. All post‐exclusion estimates (red dots) overlap with the overall effect (black vertical line), indicating no single SNP drove the association.

Building on the multivariate logistic regression results showing an inverse association between sodium intake and osteoporosis risk, we used MR to explore the potential causal relationship. The 31 SNPs selected as instrumental variables for urinary sodium all had *F*‐statistics exceeding the conventional threshold of 10 (range: 29.82–66.75), with an overall mean *F*‐statistic of 41.26, confirming the absence of weak instrument bias. As shown in Figure 5A,B and, different MR methods showed mixed but generally inverse evidence. MR‐Egger regression showed a nonsignificant estimate with a wide confidence interval (OR = 4.33, 95% CI: 0.27–69.26, *p* = 0.308), its confidence interval was broad and nonsignificant. Conversely, the weighted median (OR = 0.36, 95% CI: 0.15–0.86, *p* = 0.022) and inverse variance weighted (OR = 0.51, 95% CI: 0.28–0.95, *p* = 0.033) methods showed significant inverse associations. The scatter plot in Figure 5C suggests that the effect size of most SNPs is concentrated near the origin, but there are a few outlier SNPs (e.g., rs1260326) that exhibit large effect sizes in MR Egger regression, which may be the reason for the instability of the method estimation. The sensitivity analysis of the retention method (Figure 5D) confirmed that the overall effect estimate remained stable after the sequential exclusion of each SNP (the IVW estimate fluctuated from 0.40 to 0.55), indicating that the results were robust. Based on the results of MR analysis, this study provides a genetic basis for the negative correlation between sodium uptake and osteoporosis risk under the premise of controlling for the potential pleiotropy of genetic instrumental variables, which is consistent with the results of logistics regression studies, but the nonsignificant MR Egger regression results imply potential horizontal pleiotropy that may not have been fully ruled out. Future studies can further confirm the reliability of this association by increasing the sample size, optimizing the instrument variable selection strategy, and conducting cross‐population validation.

## 4 Discussion

Osteoporosis is characterized by low bone mass and deterioration of bone architecture, leading to increased fracture risk. The World Health Organization (WHO) clinically defines osteoporosis as BMD at least 2.5 standard deviations below the young adult reference mean (Kanis 1994). Weaver et al. (2016) reported that diet and lifestyle influence 20%–40% of adult peak bone mass (PBM), highlighting the importance of identifying dietary and lifestyle factors related to bone health.

This study comprehensively investigated the association between sodium intake and osteoporosis risk using NHANES observational data and MR analysis. Weighted logistic regression using the NHANES database revealed an inverse association between sodium intake and osteoporosis risk. This association was supported by two‐sample MR analysis (IVW: OR = 0.51, 95% CI: 0.28–0.95; *p* = 0.03).

Previous studies by Jennifer L et al. demonstrated that high sodium intake promoted urinary calcium excretion and elevated osteoporosis risk (Bedford 2011). Susie Hong et al. uncovered that sodium‐restricted diets activate the renin‐angiotensin‐aldosterone system (RAAS), and bone tissue expresses RAAS components. RAAS activation inhibits osteoblast activity, thereby increasing osteoporosis risk (Hong et al. 2022). Discrepancies in study design and population characteristics may explain these conflicting results.

In view of this, the relationship between sodium intake and the risk of osteoporosis is complex, and establishing causal inferences between the two is challenging. Therefore, beyond leveraging nationally representative observational data, our study additionally conducted MR analyses to elucidate the causal relationship between sodium intake and the risk of developing osteoporosis.

The main strengths of this study are as follows. First, the NHANES analysis included 3350 participants from a nationally representative database. Second, the MR analysis included GWAS summary data from 326,831 individuals for urinary sodium, providing complementary genetic evidence. Third, the multivariable models adjusted for a broad range of demographic, lifestyle, metabolic, and clinical covariates, which improved the robustness of the observational analysis. In addition, MR analysis can reduce confounding and reverse causation compared with purely observational studies (Zuber et al. 2022).

Although this study suggests that moderate to high sodium intake (> 6150 mg/day in the quartile‐based NHANES analysis) may be associated with lower odds of osteoporosis, this threshold should be interpreted as a study‐specific finding rather than a dietary recommendation. High sodium intake has been consistently linked to increased risk of cardiovascular diseases, including hypertension and stroke. Sodium is a well‐established modifiable risk factor for elevated blood pressure, which contributes substantially to cardiovascular morbidity and mortality. Therefore, our findings should be interpreted cautiously, considering the potential trade‐off between bone‐related outcomes and cardiovascular safety. Future dietary recommendations, if any, should be based on prospective studies and clinical trials that jointly evaluate skeletal and cardiovascular outcomes.

This study has several limitations. First, osteoporosis status was based on the questionnaire item “Have you ever been told you have osteoporosis or brittle bones?” (OSQ060) rather than BMD measurement, which is the diagnostic gold standard. Although this standardized NHANES variable has been used in population‐level epidemiological studies, it may lead to misclassification and recall bias, and it does not capture undiagnosed or BMD‐defined osteoporosis. Therefore, future studies incorporating DXA/BMD measurements are needed to validate our findings more accurately. Second, although urinary sodium is widely used as a proxy for sodium intake, we could not directly validate the correlation between dietary sodium intake and urinary sodium in the MR population because individual‐level dietary data were not available in the GWAS summary dataset. This proxy exposure may introduce measurement uncertainty. Third, although the MR‐Egger test did not reveal significant pleiotropic signals, potential residual pleiotropy remains an inherent limitation of MR studies and may bias effect estimation. Fourth, interactions between sodium intake and other dietary elements, such as calcium, magnesium, and total diet quality, are complex and could not be fully evaluated in the present analysis. Fifth, the MR analysis was based on European populations, and extrapolation to other ethnic groups should be cautious. Sixth, although our exclusion criteria addressed several major causes of secondary osteoporosis, including chronic kidney disease, parathyroid disorders, Cushing's syndrome, prolonged glucocorticoid use, autoimmune diseases requiring immunosuppression, and medications affecting bone health, we could not comprehensively exclude all causes of secondary osteoporosis, such as hyperthyroidism and chronic liver disease, because of limitations in variable availability and completeness across NHANES cycles. Residual confounding may therefore remain. Finally, the cross‐sectional design of the NHANES analysis limits causal inference. Although MR provided additional genetic evidence, prospective cohort studies and randomized controlled trials are needed to validate the relationship between sodium intake and changes in BMD and osteoporosis risk.

## 5 Conclusion

In this study, we systematically assessed the relationship between sodium intake and osteoporosis risk by combining NHANES observational data with MR analysis. In the NHANES analysis, higher sodium intake was associated with lower odds of osteoporosis in quartile‐based models (Q3 vs. Q1: OR = 0.48, 95% CI 0.30–0.78; Q4 vs. Q1: OR = 0.51, 95% CI 0.28–0.93). MR analysis using the IVW method provided genetic evidence supporting a potential inverse association (OR = 0.51, 95% CI: 0.28–0.95, *p* = 0.033), while the MR‐Egger estimate was nonsignificant. The apparent inflection points observed in the RCS analysis should be interpreted as study‐specific statistical features rather than clinically actionable intake targets. Given the established adverse effects of high sodium intake on cardiovascular health, including hypertension and stroke, these findings should be interpreted with caution and should not be translated into dietary recommendations without further evidence. Overall, our results provide hypothesis‐generating evidence for a potential relationship between sodium‐related traits and osteoporosis risk. Future prospective, experimental, and cross‐population studies are needed to validate these findings and clarify the underlying biological mechanisms.

## Author Contributions

**Xinbao Wu:** writing – review and editing, resources, funding acquisition, project administration. **Changsong Zhao:** writing – review and editing, supervision. **Zibo Fan:** writing – review and editing, methodology, visualization. **Rugang Zhao:** writing – review and editing, investigation. **Qiang Zhang:** writing – review and editing, supervision, conceptualization. **Yunxiao Ji:** conceptualization, investigation, writing – original draft, methodology, validation, visualization, writing – review and editing, software, formal analysis, data curation.

## Funding

Beijing Scholar Training Program; Beijing Municipal Public Welfare Development and Reform Pilot Project for Medical Research Institutes (code: JYY2023‐11, JYY2023‐8). National Natural Science Foundation of China (No. 82372386).

## Ethics Statement

This study used de‐identified data from the National Health and Nutrition Examination Survey (NHANES), a publicly available dataset exempt from individual consent requirements. The NHANES program has obtained ethical approval from the National Center for Health Statistics (NCHS) Institutional Review Board (IRB), and all data used in this study comply with the ethical standards set forth by the NCHS IRB. Since the data are publicly accessible and have been stripped of any personal identifiers, the need for additional ethical approval and consent from participants for this specific study was waived.

## Consent

The authors have nothing to report.

## Conflicts of Interest

The authors declare no conflicts of interest.

## Supporting information


**Table S1:** fsn372147‐sup‐0001‐TableS1.docx. *F*‐statistics of instrumental variables used in Mendelian randomization analysis.

## Data Availability

The genetic instruments for urinary sodium were obtained from the Integrative Epidemiology Unit (IEU) GWAS database, comprising summary‐level data from 326,831 European individuals. These data are publicly accessible through the IEU Open GWAS Project (https://gwas.mrcieu.ac.uk/). The observational data were extracted from the National Health and Nutrition Examination Survey (NHANES), administered by the National Center for Health Statistics (NCHS). All NHANES protocols were approved by the NCHS Research Ethics Review Board (ERB) (Protocol #2005‐06, #2011‐17, and #2018‐01), and written informed consent was obtained from all participants. NHANES data are freely available on the official website (https://www.cdc.gov/nchs/nhanes/).
