# Supplementary material for: Sodium Intake and Osteoporosis Risk: Evidence From NHANES and Mendelian Randomization Analysis
Source: Food Sci Nutr. 2026 Sep 4;14(9):e72147. doi: 10.1002/fsn3.72147 (PMC13543167; doi:10.1002/fsn3.72147)
Supplement: Supplementary file 1 [file FSN3-14-e72147-s001.docx]

| **Supplementary Table S1.**  **F-statistics of instrumental variables used in Mendelian randomization analysis** | | | | | |
| --- | --- | --- | --- | --- | --- |
| **SNP** | **Effect_allele** | **Qther_allele** | **Beta** | **SE** | **F** |
| rs1194277 | G | C | 0.014715 | 0.002382 | 38.17498 |
| rs13383034 | T | C | 0.014552 | 0.002572 | 32.00152 |
| rs1260326 | C | T | -0.01953 | 0.002418 | 65.23197 |
| rs1516187 | C | T | 0.020952 | 0.003496 | 35.90854 |
| rs1437971 | C | A | -0.01684 | 0.002509 | 45.06508 |
| rs11706708 | T | C | 0.018598 | 0.002895 | 41.25466 |
| rs7442885 | G | C | -0.01943 | 0.002911 | 44.55631 |
| rs13188076 | T | G | -0.01592 | 0.002804 | 32.21826 |
| rs2754809 | G | C | 0.014558 | 0.002602 | 31.29822 |
| rs12189679 | A | G | -0.01589 | 0.002373 | 44.83484 |
| rs6900444 | T | C | 0.013362 | 0.002382 | 31.47039 |
| rs1327285 | G | A | -0.0224 | 0.002791 | 64.44065 |
| rs4410790 | C | T | -0.01687 | 0.002456 | 47.17015 |
| rs7800944 | C | T | -0.01632 | 0.002615 | 38.95527 |
| rs62466423 | T | C | -0.02403 | 0.004248 | 32.00971 |
| rs55861011 | T | C | -0.01392 | 0.002548 | 29.85667 |
| rs140154612 | C | T | -0.05677 | 0.009511 | 35.62702 |
| rs11103388 | A | G | 0.014963 | 0.002518 | 35.31394 |
| rs2393831 | G | A | -0.01559 | 0.002845 | 30.02693 |
| rs2807978 | C | T | -0.01716 | 0.002616 | 43.03578 |
| rs7924036 | T | G | -0.01306 | 0.002367 | 30.43702 |
| rs2210143 | G | T | -0.01694 | 0.00254 | 44.4538 |
| rs2945091 | A | G | -0.01421 | 0.002379 | 35.68202 |
| rs569770 | C | T | 0.013742 | 0.002469 | 30.98977 |
| rs12581220 | T | C | -0.01797 | 0.002554 | 49.48919 |
| rs1957111 | T | C | 0.033688 | 0.005363 | 39.45562 |
| rs2472297 | T | C | -0.02029 | 0.00267 | 57.75254 |
| rs11642015 | T | C | 0.017926 | 0.002412 | 55.22799 |
| rs12936340 | A | G | 0.013564 | 0.002465 | 30.28855 |
| rs784257 | C | T | 0.016633 | 0.003046 | 29.82395 |
| rs8097672 | T | A | 0.01871 | 0.003368 | 30.85243 |
| rs28399462 | A | G | 0.051991 | 0.008006 | 42.17452 |
| rs34783010 | T | G | -0.0217 | 0.002987 | 52.75361 |
| rs838133 | G | A | 0.019996 | 0.002447 | 66.75405 |
